# TAR RNA Mediated Folding of a Single-Arginine-Mutant HIV-1 Tat Protein within HeLa Cells Experiencing Intracellular Crowding

**DOI:** 10.3390/ijms22189998

**Published:** 2021-09-16

**Authors:** Jung Min Kim, Honggu Chun

**Affiliations:** Division of Interdisciplinary Program in Precision Public Health (BK21 FOUR Program), Department of Biomedical Engineering, Korea University 145 Anam-ro, Seongbuk-gu, Seoul 02842, Korea; erine7.kim@gmail.com

**Keywords:** crowding effects, chaperna, folding kinetics, TAR RNA, Tat, arginine-rich domains

## Abstract

The various effects of native protein folding on the stability and folding rate of intrinsically disordered proteins (IDPs) in crowded intracellular environments are important in biomedicine. Although most studies on protein folding have been conducted in vitro, providing valuable insights, studies on protein folding in crowded intracellular environments are scarce. This study aimed to explore the effects of intracellular molecular crowding on the folding of mutant transactivator HIV-1 Tat based on intracellular interactions, including TAR RNA, as proof of the previously reported chaperna-RNA concept. Considering that the Tat–TAR RNA motif binds RNA, we assessed the po tential function of TAR RNA as a chaperna for the refolding of R52Tat, a mutant in which the argi nine (R) residues at R52 have been replaced with alanine (A) by site-directed mutagenesis. We mon itored Tat-EGFP and Tat folding in HeLa cells via time-lapse fluorescence microscopy and biolayer interferometry using EGFP fusion as an indicator for folding status. These results show that the refolding of R52A Tat was stimulated well at a 0.3 μM TAR RNA concentration; wild-type Tat refolding was essentially abolished because of a reduction in the affinity for TAR RNA at that con centration. The folding and refolding of R52Tat were mainly promoted upon stimulation with TAR RNA. Our findings provide novel insights into the therapeutic potential of chaperna-mediated fold ing through the examination of as-yet-unexplored RNA-mediated protein folding as well as viral genetic variants that modulate viral evolutionary linkages for viral diseases inside a crowded intra cellular environment.

## 1. Introduction

In the intracellular environment, the structures and functions of various proteins exhibit cooperative interactions under crowded conditions, with particular folding energies and kinetics [1,2,3]. The relative resistance of intrinsically disordered proteins (IDPs) to forming oligomers in their folded structures can be detrimental to cells under crowded conditions [4,5,6] because it is sensitive to the effects of cellular crowding [5].

To date, most protein-folding studies have focused on in vitro analyses and have not considered the sensitive intracellular molecular interactions induced by intracellular crowding [3,7,8,9,10,11]. Several studies have used crowding agents to induce conformational changes in unfolded proteins [12]. For example, cytochrome c molecules in unfolded and molten globular states have been found in two essentially unstructured proteins in the presence of crowding agents at low pHs [11,13,14,15]. Some in vitro studies have reported the effects of high concentrations of macromolecules, including complex proteins, demonstrating the effects of macromolecular crowding agents on the folded structures of small, single-domain proteins under physiological conditions [16]. To date, model systems, such as cancer cell lines or bacteria, have been utilized to directly study protein folding inside cells and analyze it via in-cell NMR or fluorescence microscopy [17,18,19].

We have demonstrated that TAR RNA allows chaperone function in a single arginine mutant, R52Tat, under conditions of HeLa cell crowding. The HIV-1 transactivator protein Tat is essential for the high-level expression of the HIV TAR element and requires transcription by RNA-acting regulatory elements [20,21,22]. When Tat binds to P-TEFb in the absence of TAR RNA, the TAR RNA binding region remains unstructured [23,24]. The virus-encoded TAR RNA and ligand–Tat interaction with other cellular proteins exert intrinsic effects on the activation of transcription. Therefore, the presence of a single arginine 52 (R52) residue within mutant Tat enables transcription to produce a common structural motif in the TAR of the region of the viral genome [25]. Several studies have attempted to elucidate the mechanism underlying the effects of this arginine region on protein aggregation and protein misfolding [25,26,27,28,29,30]. 

The intracellular environment shows the structures and functions of various proteins under crowding effects on the energy and kinetic influence of protein folding. The crowding is an extremely complicated intracellular environment with a confined amount of free water and an almost complete lack of unoccupied space [3,8,13,31]. This reported tendency for the excluded volume minimization in crowding is expected to affect proteins and is related to and driven by the need of a system to maximize its entropy [3,13,31].

Several studies have shown that macromolecular aggregation can affect protein structure, folding, shape, conformational stability, small molecule binding, enzymatic activity, protein–protein interactions, protein–nucleic acid interactions, and pathological aggregation.

**Figure 1:**
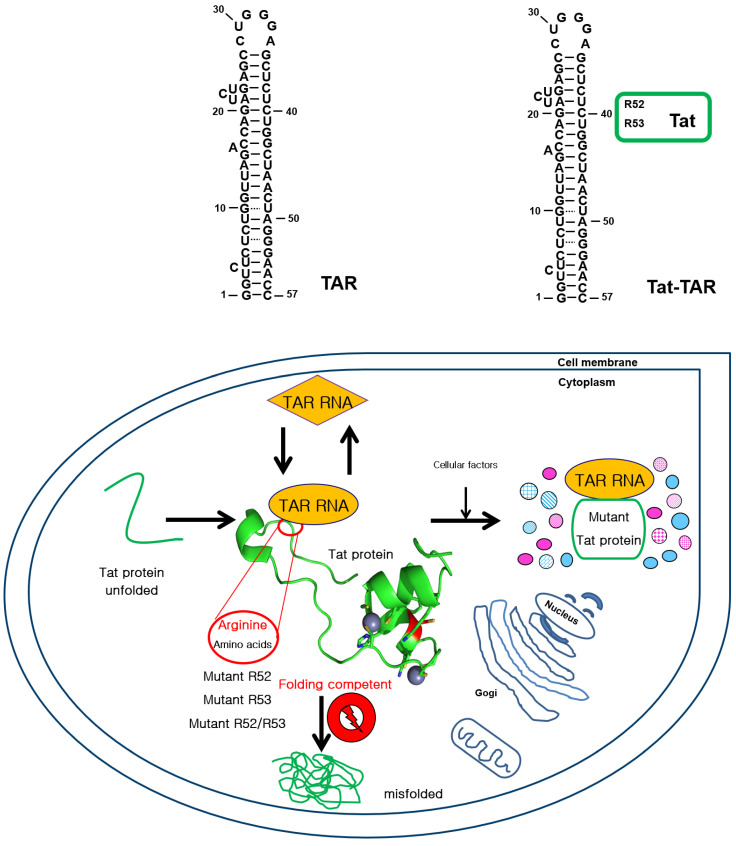
The TAR RNA sequence and chaperna for a single-arginine residue mutant Tat refolding. This figure shows TAR RNA as a molecular chaperone for mutant Tat protein. Tat binds to TAR, which is a highly structured element that is ‘folding competent’ and that specifically interacts with arginine residues. The TAR RNA sequence originates from the complete genome of HIV-1. It comprises 57 base pairs and a stem–loop structure. Two selected arginine residues, R52 and R53, were each selected from the HIV Tat protein for single-point mutation by substitution with alanine. All the mutants of Tat can interact with cellular factors—e.g., cyclin T1 (pink) and P-TEFb (blue)—and can be further regulated by its TAR RNA.

Previous studies have suggested that binding to TAR RNA requires R52Tat to be soluble [32]. Soluble Tat mutants replace the arginines at positions 52 and 53 (R52 and R53), which are critical for binding to TAR RNA. Arginine-rich motifs within Tat recognize TAR RNA, suggesting the relative importance of specific amino acids within RNA-binding motifs. The viral RNA-synthesis transactivator Tat protein of HIV-1 belongs to a large family of IDPs. The single arginine 52 (R52) region within R52Tat strongly stimulates TAR RNA in uncrowded conditions. These results suggest that arginine residues in the conserved region of Tat affect the solubility, folding, and RNA-binding ability of interacting proteins. Building on these earlier studies, we investigated the mutation of a single arginine residue in the conserved basic region of Tat via site-directed mutagenesis to evaluate the effect of TAR RNA binding on the kinetics of R52Tat refolding under crowded conditions in HeLa cells. Nagai et al. have reported arginine as a potential new means with which to assess the molecular mechanisms and symptoms of weakness in polyQ disease [33]. In this study, we sought to explore the importance of intracellular molecular crowding in protein folding by considering various intracellular interactions, including enzymes and RNA-mediated protein-folding substrates (Figure 1). 

RNA molecules play a central role and have functions in virtually all cellular processes. There are various ways in which RNA can have chaperoning effects on bound proteins. RNA-facilitated protein folding is closely associated with problems in protein folding and with both protein structure and the mechanisms by which proteins achieve their active conformations [34]. The protein chaperone aids in RNA folding, and chaperonins mainly assist with RNA folding in a passive manner. This indicates that their function in RNA folding occurs through direct contact and interaction with exposed hydrophobic residues in nonnative proteins [35,36]. This mechanism could increase, rather than decrease, the folding rate of RNA-bound proteins. All newly synthesized polypeptides interact with RNAs, including mRNA, during the folding process [37,38,39,40]. Natively unfolded or partially disordered proteins switch to folded or ordered structures upon contact with their cognate RNAs [38,41,42,43,44]. How can functional RNAs be distinguished from misfolded protein intermediates? Chaperones and chaperna-RNAs that function as chaperones mainly assist in protein folding in a passive manner, meaning that they assist through direct interaction with exposed hydrophobic residues on nonnative proteins [45,46,47]. The role of traditional molecular chaperones in protein folding is limited, and they cannot participate in a wide range of protein-folding processes; thus, other crucial chaperone types that have been overlooked must exist [48]. In this context, the role of RNAs in the folding and aggregation of the proteins with which they interact has to be determined and understood.

The presence of hydrophobic residues, such as alanine, on the interior of a mutant Tat with reduced refolding ability did not lead to a further reduction in this ability, indicating that a single arginine residue is associated with R52Tat-binding TAR RNA. A single arginine residue is associated with mutant and was constructed under the same concentration of TAR RNA that assists in mutant Tat refolding, and it was compared with wild-type Tat. These results show that the arginine residue in the conserved basic region of R52Tat is crucial for binding TAR RNA and that the affinity of this RNA may also influence the refolding and folding of its interacting partner, R52Tat.

## 2. Results

### 2.1. Folding of Single-Arginine-Residue-Mutant Tat with TAR RNA

To identify the amino-acid associations that are important for TAR RNA recognition within the RNA sequence, the affinity and specificity of TAR for residues 47–58 of the arginine-rich region of mutant Tat were plotted (Figure 1). The HIV Tat protein has a highly conserved basic region that contains two lysine and six arginine residues among nine amino acids; this region mediates specific binding to TAR RNA. The distal part of the TAR RNA contains 57-base stem–loop structures (Figure 1). The Tat protein structure suggests that the arginine-rich RNA-binding domains are unstructured when they are similarly unbound to RNA and can be fully or partially structured upon binding to interact with RNA. Not all the positions in the sequence are equally important for Tat activity and Tat–TAR affinity because single substitutions of arginine residues at positions 52 and 53 have slightly greater effects than substitutions of arginine residues at positions 55, 56, and 47–58. Therefore, we selected R52 and R53 to create single-residue mutations with substitutions for alanine. The nucleotides marked with green circles (R52 and R53) make up the TAR RNA binding region of the Tat protein (Figure 1). 

We examined whether TAR RNAs assisted in folding when bound to the arginine-rich domain of the Tat protein in the cytoplasm. Through ionic interactions between the arginines at positions 52 and 53, the Tat amino groups are arranged in a particular tertiary form; Tat and TAR RNA are RNA-phosphate-binding regions where Tat transactivation occurs. These arginine residues at positions 52 and 53 in Tat are in a specific RNA-binding region where Tat transactivation occurs through the ionic interaction between the amino group of arginine and the RNA phosphate, which are arranged in a specific tertiary form as a ‘specific affinity’ contact between Tat and TAR RNA. 

In this study, we introduced a mutation into Tat at the binding site within the TAR RNA sequence to demonstrate that chaperna–TAR RNA binding is important for protein refolding inside the cell. The mutation of some protein residues influences the conformation and alters the protein structure. These results imply that the single arginine residue within Tat is associated with protein folding because hydrophobic residues, such as alanine, tend to be located on the internal surfaces of proteins. Additionally, the diminished refolding of the mutant Tat did not lead to a decreased ability to bind TAR RNA. Thus, a TAR RNA binding mutant protein was constructed using site-directed arginine mutagenesis and compared with wild-type Tat.

### 2.2. Validation of EGFP Unfolding and Refolding In Vitro and upon HeLa Cell Crowding

**Figure 2:**
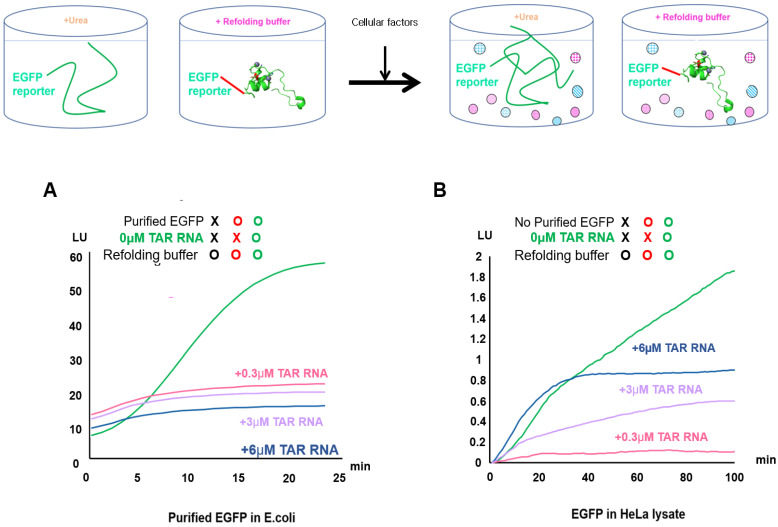
Kinetic refolding of EGFP as a folding reporter under crowded or uncrowded conditions. The effect of refolding at different TAR RNA concentrations on the EGFP (green line) from HeLa lysate and the EGFP (green line) from *E. coli* was analyzed. (**A**) The EGFP intensity of refolding in uncrowded conditions was measured from EGFP refolding at specific concentrations (0.3, 3, and 6 µM) of TAR RNA for 25 min (*E. coli*), with a refolding time of 10 min. (**B**) The effects of uncrowded conditions compared with the effects of crowded conditions on the GFP intensity were measured based on EGFP refolding at specific concentrations (0.3, 3, and 6 µM) of TAR RNA for 80 min (HeLa lysate), with a refolding time of 40 min. The fluorescence intensity of the EGFP emission is given in relative light units (LU).

The main difference between the refolding experiments using *E. coli* (non-crowded solution) and those conducted using crowded HeLa conditions was the influence of vari ous intracellular factors (Figure 2). EGFP was used in our study as a single-cell expression reporter to allow a substantial distinction between correctly folded and aggregated target proteins in live *E. coli* and mammalian cells (Figures S1–S4). In this experiment, non-crowded (purified protein) and crowded conditions in *E. coli* were compared during in vitro refolding to determine whether EGFP enhanced refolding under conditions featuring intracellular crowding in HeLa lysates (Figure S5B and Figure 2). We found differences in the initial EGFP refolding rate between the intracellular crowded conditions in HeLa lysate and non-crowded conditions in *E. coli*.

Previous reports have described in vitro studies of EGFP protein unfolding and refolding to understand how proteins fold inside cells [49,50]. We investigated the mechanism underlying the EGFP reporter’s promotion of protein folding via enzymes and protein-folding substrates in crowded solutions. We compared EGFP from HeLa lysate and purified EGFP from *E. coli*, as well as wild-type EGFP from the HeLa lysate and purified EGFP (Figure S5A) from *E. coli* treated with 6 M urea. The purified EGFP from *E. coli* was completely inactivated by treatment with 6 M urea; the non-purified green fluorescence in the HeLa lysates gradually disappeared when they were treated with 6 M urea (t1⁄2: 80 min; Figure S5A). By contrast, the purified green fluorescence in *E. coli* disappeared after 20 min at high speed (Figure S5A). To distinguish between these effects, we measured the rate and yield of EGFP refolding by monitoring the increase in intrinsic EGFP fluorescence under refolding conditions (Figure 2B). The EGFP fluorescence intensity was not significantly attenuated under refolding conditions with TAR RNA treatments (0.3, 3, and 6 µM). The fluorescence intensity of purified EGFP in *E. coli* increased in a time-dependent manner to approximately 90% of that of the refolded EGFP, as measured under EGFP refolding conditions at 30 s intervals for 30 min (Figure 2A). By contrast, EGFP from HeLa lysates showed a linear increase in the intensity of the fluorescence from the denatured EGFP under refolding conditions at 1 min intervals for 3 h (Figure 2B and Figure S5B).

### 2.3. Effect of TAR RNA as a Helper in Refolding Mutant R52Tat-EGFP Involves Enzymes and Protein-Folding Substrates under Crowded HeLa Conditions

**Figure 3:**
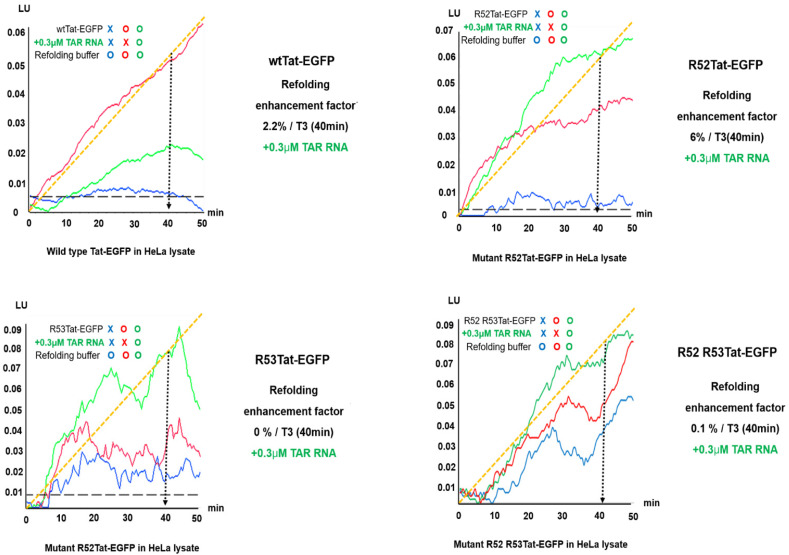
Comparative kinetic analysis of the refolding of mutants R52, R53, and R52R53Tat-EGFP in the presence of TAR RNA. The refolding enhancing factor was measured via the fluorescence of wild-type/mutant R52Tat–EGFP in the presence of 0.3 μM TAR RNA in HeLa lysates. The increase in the fluorescence intensity of EGFP was monitored by the time-dependent increases in the fluorescence of wild-type Tat-EGFP and mutant Tat-EGFP. In the presence of 0.3 µM TAR, it was possible to observe refolding enhancement factors over the time course of the fluorescence intensity. A refolding enhancing factor for R52Tat fusion EGFP in the absence or presence of TAR RNA in 0.3 µM HeLa cell lysate of ~6% at 40 min is shown by the green line. Refolding enhancing factors of R53 and R52R53Tat fusion EGFP in the absence or presence of TAR RNA in 0.3 µM HeLa cell lysate of ~1% and 0% at 40 min are shown by the green line. The refolding enhancing factor of wild-type Tat fusion EGFP in the absence or presence of TAR RNA in 0.3 µM HeLa cell lysate of ~2.2% at 40 min is shown by the green line. All the data are the averages of duplicate experiments. Color plots represent the data ranges via linear fitting. EGFP emissions are given in relative light units (LU). Refolding buffer baselines were subtracted from all the data.

**Figure 4:**
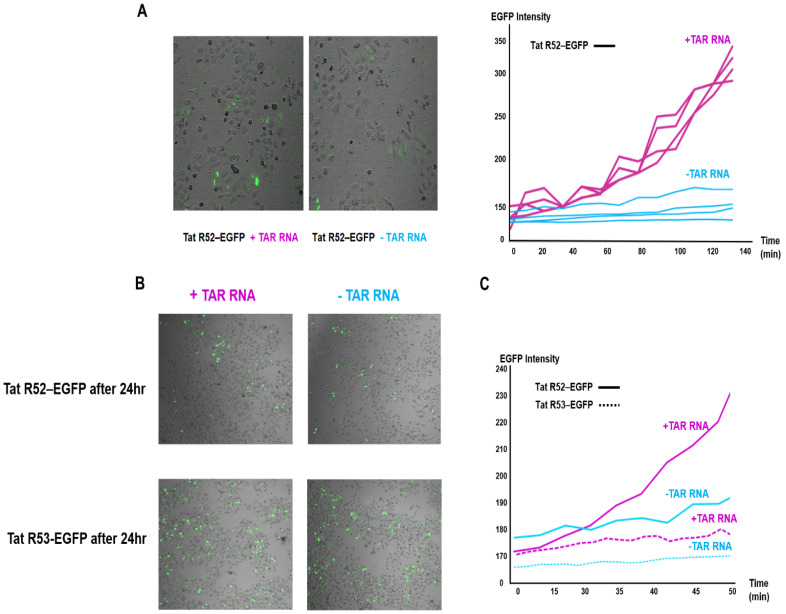
Live time-course-dependent changes in fluorescence in mutant Tat folding with TAR RNA in HeLa cells were monitored. The relative proportions of single-arginine-mutant R52 and mutant R53Tat-EGFP expressed in live cells expressing EGFP. Time-dependent increases in R52Tat-EGFP are shown using a scatterplot of the regions of interest (ROIs) corresponding to Videos S1 and S2 of R52 and R53Tat-EGFP fluorescence in the control (blue lines) and TAR RNA co-expressed (red lines) cells. Time-lapse images of R52Tat-EGFP expression in HeLa cells at four independent positions were captured. The mean fluorescence of ROIs was traced using differential interference contrast (DIC) covering the entire numbering field. After transfection and 5 h of incubation, HeLa cells were co-transfected with plasmids encoding mutant R52, R53Tat-EGFP, and TAR RNA and observed under live cell fluorescence microscopy at 10 min intervals for 2 h. (**A**) Merged images (40X, left) of the DIC and the fluorescence (EGFP) 3 h after transfection. Graphical presentation of the time-dependent increases in R52Tat-EGFP (linear line) expression in live HeLa cells. (**B**) Overlaid image (10X) used to generate the numbers of R52Tat-EGFP (linear line)- and R53Tat-EGFP (dot line)-expressing cells 24 h after transfection. All scale bars are 200 µm. (**C**) Comparison of the time-lapse fluorescence observations of R52 and R53Tat-EGFP expression with TAR RNA (red lines) and without TAR RNA co-expression (blue lines).

To monitor the unfolding and refolding of the HIV Tat protein in vitro, EGFP was fused to the C-terminus of the Tat protein (to make Tat-EGFP). We measured the enhancement of Tat-EGFP refolding by monitoring the increase in intrinsic EGFP fluorescence under denaturing Tat-EGFP and folding conditions (Figure 3 and Figure 4). As shown in Figure 3, the wild-type (wt) Tat-EGFP’s fluorescence intensity did not show any increase in refolding enhancement under refolding conditions following treatment with TAR RNA (0.3 μM).

The influence of TAR RNA on the folding of wtTat-EGFP in vitro was further investigated by employing TAR RNA at various concentrations (0.3, 3, and 6 µM; data not shown). In the presence of TAR RNA, the refolding enhancement factors for EGFP (Figure 3 and Table S1) were significantly lower than those for wtTat-EGFP. These results (Figure 3) indicate that it is difficult to directly monitor the proper folding of wtTat protein without EGFP and that TAR at a concentration of 0.3 μM is sufficient to support wtTat-EGFP protein refolding. Figure 2B shows the TAR RNA mediated refolding of EGFP from HeLa lysate in vitro. However, the fluorescence signal indicative of wtTat-EGFP refolding was dependent on the TAR RNA concentration in the HeLa cell lysate; Tat-EGFP assays of HeLa cell extracts at 0.3 and 3 µM TAR RNA showed a wtTat-EGFP refolding enhancement factor of zero (data not shown). These results strongly support the suitability of EGFP as a refolding reporter for assessing the helper function of TAR RNA for wtTat- EGFP protein refolding in HeLa cells. Therefore, we confirmed the possibility that the signal intensity of EGFP alone or refolded wtTat-EGFP decreased at 0.3 and 3 µM concentrations of TAR RNA in HeLa lysate. 

Wild-type Tat-EGFP, TatR52A-EGFP, and TatR53A-EGFP were used to assess the unfolding and refolding of mutant Tat proteins (Figure 3). The mutations of the arginine residues, R52A and R53A, in the conserved basic region of Tat are crucial for specific TAR RNA binding and the enhanced refolding of the R52 mutant Tat. Next, we expressed wild-type Tat-EGFP, TatR52A-EGFP, TatR53A-EGFP, and TatR52AR53A-EGFP in HeLa lysate. The refolding enhancement factors for TatR52A-EGFP increased, as measured by the EGFP response, during a refolding period of 1 h in the absence and presence of TAR RNA at the optimized concentration (0.3 µM; Figure 3). Following this, we determined the effects of *E. coli* cells in an uncrowded environment on (purified) EGFP during a refolding period of 20 min and compared the results with the effect of a crowded environment in HeLa lysate during a refolding period of 1 h to normalize the measurement of wild-type Tat-EGFP to the EGFP response (Figure 2 and Figure 3). 

The refolding yields for TatR52A-EGFP and TatR52AR53A-EGFP at the optimized TAR RNA concentration (0.3 µM) were significantly higher than those for wild-type Tat-EGFP in HeLa lysate (Figure 3). These data show that the mutations affected the interac tions between TAR RNA and mutant Tat wild-type Tat protein, suggesting that direct interaction with TAR RNA was important for promoting TAR RNA mediated R52 mutant Tat protein refolding in HeLa lysate. The refolding enhancement of the R52Tat proteins was generally higher than that of the wtTat.

The refolding enhancement factor for mutant R52ATat-EGFP was 6% in 0.3 µM TAR RNA, whereas that for wild-type Tat was 0% (Figure 3). The yield of R52 mutant Tat-EGFP refolding was proportional to the concentration of TAR RNA at 0.3 µM, whereas the re folding of wild-type Tat was absent (Figure 3). Table S1 shows the refolding enhancement factor in a detailed analysis of the refolding of the Tat protein. The R52Tat has been shown to be important for the recognition and binding of TAR RNA, reflecting the folding of GFP, including autocatalytic cyclization and oxidation reactions for chromophore formation. Additionally, the relevant single arginine within the motif has been determined. These results indicate that TatR52A-EGFP and TatR53A-EGFP are slightly prone to aggregation in the absence of TAR RNA. In addition, we suggest that the misfolding of TatR52A-EGFP can be prevented by the presence of 0.3 µM TAR RNA.

### 2.4. Increased Participation of Single-Arginine Mutants R52 and R53 in Tat-EGFP Folding Mediated by TAR RNA in Live Cells

To investigate the chaperone function of TAR RNA for the arginine-mutant Tat-EGFP protein, the folding of mutant Tat-EGFP in live HeLa in the presence or absence of TAR RNA was tested (Figure 4). 

Previous studies have shown that fluorescence signals from the expression of EGFP alone were not affected by RNA co-expression; hence, a control was used [32,51]. HeLa cells were cotransfected with single-residue-mutant Tat-EGFP and TAR RNA, and the fluorescent cells were traced and monitored using live cell time-lapse fluorescence microscopy (Figure 4A,C). After transfection and 5 h of incubation, the initial rates and yields of the folding of the R52 and R53Tat-EGFP mutants were estimated by monitoring the increase in the intrinsic EGFP intensity. The temporal change in the fluorescence of the R52Tat-EGFP mutant was monitored every 10 min for 2 h by tracking regions of interest (ROIs, matched to the fluorescence of the cell) in the videos; it is graphically represented in Figure 4B. The level of fluctuation in the measured fluorescence signal of the mutant Tat-EGFP reflects the degree of difficulty in tracking live, migrating single cells. 

Given the use of live cell time-dependent fluorescence monitoring, cytoplasmic EGFP fluorescence was used to indicate the appropriate folding of the R52 (Videos S1 and S2) and R53Tat mutants, which was quantified using the measured mean fluorescence of the ROIs with TAR RNA treatment. The expression of the EGFP reporter showed an increase in the number of fluorescent cells 24 h after cotransfection with the mutant Tat-EGFP and TAR RNA plasmids compared to the TAR RNA only controls (Figure 4B and Figure S2). Much of the punctate localization observed by fluorescence microscopy corresponded to the presence of wild-type Tat-EGFP and TatR52A-EGFP in nuclear speckles of varying sizes and irregular shapes (Figures S2 and S3). In these cells, wild-type HIV Tat-EGFP was localized in a diffuse pattern throughout the nucleus, including in the nucleolar regions, with some foci of higher accumulation indicative of speckles in HeLa cells. As previously reported, Tat is located in the nucleolus and nucleosome accumulation occurs through a stretch of highly conserved basic amino acids that serve as nucleosome position signals (NoLSs) [52,53]. The arginine/serine-rich domain (RS domain) is necessary and sufficient for the formation of nuclear speckles. The arginine residues in this motif play a key role in RNA interactions, which studies have reported as being sequence-independent but necessary for RNA export [54,55]. The basic region of Tat has been identified as a NoLS and targets Tat to the nucleolus.

These reports are consistent with the behavior of the transdominant Tat mutant, which is confined to the nucleus but not to the nucleolus. In addition, TAR RNA like protrusions are highly structured in nucleosomes, suggesting that they are present in synthesized ribosomal RNA. Interestingly, some ribosomal proteins, such as Tat, contain arginine-rich regions, which have been proposed to mediate specific RNA binding. A vector expressing only the EGFP tag was used as a control; proteins were uniformly distributed throughout the cell and did not colocalize with speckles in HeLa cells, whereas speckles were identified in Tat-EGFP.

Nevertheless, the average EGFP fluorescence intensity, considering the statistical significance of signals from single EGFP cells, indicated the notable stimulation of mutant R52Tat-EGFP signals in the presence of TAR RNA (Figure 4C). The enhancement of both the initial rate and fluorescence intensity of the mutant R52Tat is consistent with the potential chaperoning role of TAR RNA in the folding of the mutant R52Tat in live cells.

### 2.5. Optimization of Biolayer Interferometry Monitoring to Analyze Arginine-Mutant Tat Refolding without EGFP

**Figure 5:**
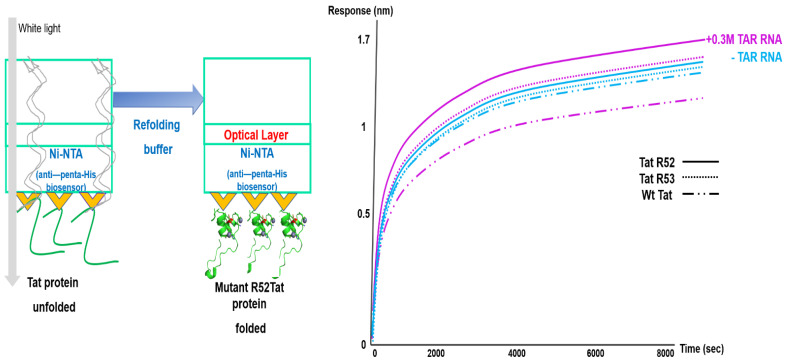
Comparative kinetic analysis of refolding using biolayer interferometry with mutant R52Tat in the presence of TAR RNA. The effects of mutant R52Tat on the refolding yield in the presence of 0.3 µM TAR RNA within HeLa lysates. Immobilized His-tagged wild-type and mutant Tat refolding was assessed using an anti-penta-His biosensor on an Octect RED 96 system by monitoring the function of chaperna through a TAR RNA assay in HeLa lysates. A representative BLI color sensogram is shown, indicating a preferential increase in mutant Tat refolding in the presence of TAR RNA (red lines) relative to wild-type Tat refolding, which is related to the hydrophobic surfaces as chaperna progresses. The refolding of wild-type Tat (middle dotted line), TatR52 (solid line), and TatR53 (dotted line) in the absence of TAR RNA (blue lines) and the refolding of wild-type Tat (middle dotted line), TatR52 (solid line), and TatR53 (dot line) in the presence of 0.3 µM TAR RNA (red lines) are shown (specific binding to anti-penta-His; left *y*-axis). All the data are the average of duplicate experiments. Color plots represent the data range. Buffer baselines were subtracted from all the data.

To optimize the refolding of mutant R52Tat without EGFP, we compared the effect of an uncrowded environment on the TatR52A (purified) in *E. coli* cells with that of a crowded environment on EGFP in the absence of TAR RNA by BLI at 1.3 nm during a refolding period of 20 min (Figures S4 and S5) [56]. We used this well-characterized system to assess the response of His-tagged Tat-BLI after binding to the TAR RNA substrate to design a BLI-detection study for mutant R52Tat folding without EGFP in the absence of TAR RNA (Figure 5 and Figure S4) [3,57]. To normalize the refolding efficiency of the R52 mutant Tat, we monitored refolding buffer alone as a negative control. The immobilized His-tagged R52 and R53Tat in the refolding buffer enabled the identification of superior refolding or aggregate formation and the effects on chaperna function in HeLa lysate. The treatment of the denatured mutant Tat with TAR RNA led to an initial increase in the BLI response at 1.6 nm during the refolding period of 2 h (Figure 5). These results support our hypothesis that TAR RNA is required for refolding TatR52A and TatR53A.

Immobilized His-tagged TatR52 and TatR53 in refolding buffer can identify superior refolding conditions by rapidly detecting aggregate formation and affecting the function of chaperna in HeLa lysate. As shown in Figure 5, we observed that TAR RNA binding decreased with the refolding of immobilized His-tagged wild-type Tat protein (binding specifically to anti-penta His; left *y*-axis). By contrast, TAR RNA binding with refolding increased the levels of immobilized His-tagged TatR52A and TatR53A proteins. The treatment of the denatured mutant Tat-TAR RNA led to an initial increase in the BLI response at 1.6 nm during the 2 h refolding period. These results closely resemble those obtained using TAR RNA-mediated TatR52A-EGFP and TatR53A-EGFP refolding. This similarity was also noted in TatR52A and TatR53A refolding without EGFP. Figure 5 shows that the refolding of TatR52A and TatR53A was mediated by TAR RNA. Therefore, our results support our hypothesis that TAR RNA is required for refolding TatR52A and TatR53A.

## 3. Discussion

We examined the chaperna-RNA that functions as a chaperone for Tat proteins in the folding of the single-arginine mutants TatR52 and R53. The RNA chaperone was distinguished from proteins that aid in the folding of interacting RNA; here, we propose the use of an RNA-based chaperone that aids in the folding of interacting proteins. In this study, TAR RNA, as a chaperna, showed strong evidence of a folding kinetics model being applicable. This demonstrated its contribution under crowded macromolecular conditions in living HeLa cells. Thus, TAR RNA has chaperone activity and dictates the folding of the mutant R52Tat. In addition, TAR RNA affected the kinetics of Tat folding in crowded intracellular conditions. The present results are consistent with the prediction that TAR RNA interacts with arginine residues in the basic conserved region of Tat under the conditions of intracellular crowding to increase R52Tat refolding. 

Despite Tat’s inherently unfolded nature, little is known about how the incorrect folding of the Tat protein can be prevented inside cells. The Tat protein has the potential to interact with several host proteins and fold into a stable form in a transactivator complex [58]. However, the maintenance of its folding ability remains unknown. This ability plays a pivotal role in HIV transactivation by mobilizing host proteins to the stem–bulge–loop structure of the viral mRNA of TAR [59]. The deletion of this Tat region significantly reduces Tat transactivation; therefore, it is hypothesized that nucleolar localization is required for the transactivation of Tat function [60,61,62,63]. The nuclear accumulation of proteins can be attributed to the interaction of long basic amino acids with ionic sites such as RNA-binding nuclear proteins [53,64]. These results suggest that the arginine-rich motifs that characterize viral proteins tend to be disordered and partially aligned when the RNA-binding domain has a significant sequence flexibility that enables it to interact with cognate RNA and reversibly interacts with the abundant RNA inside the cell.

To confirm RNA-mediated integrative folding, we rapidly monitored the proper folding of Tat protein in the presence of TAR RNA for enhanced green fluorescent protein (EGFP) fusion to the C-terminus of Tat with EGFP. The refolding of R52Tat was promoted upon stimulation with TAR RNA under crowded conditions. Live cell imaging also showed the extent to which the folding rate of mutant R52 and R53Tat-EGFP was stimulated by TAR RNA. 

Considering that the main function of molecular chaperones undergoing intermolecular electrostatic repulsion by cell densification is to prevent cellular protein folding and the aggregation of unfolded proteins, the assay was related to chaperone activity.

The core principle described here can be applied to various cellular-swelling- or shrinkage-activated responses that involve enzymes and RNA-mediated protein-folding substrates and can stimulate protein folding via intermolecular repulsion. Thus, protein conformational changes, conformational entropy, and the unstable intermolecular repulsion forces of cellular macromolecules that are distinct (but fully compatible) from attractive interactions need to be considered in crowded cellular environments [65]. 

In particular, mutations in charged residues that reduce intermolecular electrostatic repulsion are implicated in aggregation-associated diseases. The general concept of molecular chaperones introduces auxiliary protein folding instead of spontaneous protein folding. It has been suggested that cellular macromolecules, including chaperones, exhibit an intrinsic chaperone activity that prevents the aggregation of physically linked polypeptides regardless of the type of linkage between them, usually due to their large exclusion volumes and intermolecular repulsion due to surface charges. However, genetic and biochemical analyses have shown that only a limited number of proteins are folded with the help of molecular chaperones [66,67,68,69]. Protein folding results in living cells that may significantly differ from those obtained under controlled in vitro conditions in terms of the cell biochemical ratios and molecular equilibrium conditions.

Thus, in various cell-dense environments, proteins can be greatly affected by the intermolecular repulsive forces of bulky cellular macromolecules that physically interact with the proteins. A more compact state may be thermodynamically favored, such as the folded and aggregated structures of dense cellular macromolecules. To date, proteins in cell-dense environments have largely been discussed as experiencing volume effects excluded from indirect stabilization by macromolecular clustering that do not involve physical linkages between proteins and cellular macromolecules. Evidence that macromolecular aggregation is a real intracellular phenomenon comes from the differences in protein-folding-rate measurements obtained in purified in vitro conditions in terms of the cell biochemical ratios and molecular equilibrium conditions in living cells (Figure 3 and Figure S4).

Along with the protein-folding rate and thermodynamic stability, protein conformation is an important determinant of protein aggregation. Anfinsen’s thermodynamic hypothesis relates to the concept of protein folding, which is the intrinsic structure of a protein that is the most stable under physiological conditions and, thus, occurs spontaneously [70]. However, since intramolecular forces are independent, a high thermodynamic stability alone cannot guarantee the prevention of agglomeration [71]. For example, native protein structures are thermodynamically unstable to amyloid fibrils or non-amyloidogenic aggregates in their bound protein-folding and aggregation environment, which is suggested to be involved in aggregation-related neurodegenerative diseases [72,73]. Therefore, in addition to proper protein-folding and thermodynamic stability, the surface charge of proteins, chaperone-assisted protein-folding capacity, and protein-folding should be excluded from protein-folding thermodynamics, which challenges the thermodynamic hypothesis of Anfinsen.

Tat has been shown to exhibit RNA chaperone activity, which aids in the folding and structural rearrangement of RNA molecules and is possibly mediated by interactions with essentially disordered regions [74]. The deletion of this Tat region significantly reduces Tat transactivation; therefore, it is hypothesized that nucleolar localization is required for Tat’s functional transactivation [60,61,62,63]. The nuclear accumulation of proteins can be attributed to the interaction of long basic amino acids with ionic sites such as RNA-binding nuclear proteins [53,64]. Alternatively, other nuclear components, such as ribosomal proteins, may be the binding sites for these “nuclear targeting” sequences [75]. 

RNA contains negative charges that cause electrostatic repulsion and block intermolecular interactions that lead to aggregation. These properties can stabilize intermediate folding and interfere with protein aggregation. This is particularly interesting considering previous reports that the single arginine region of R52Tat itself has been shown to aid in the folding and conformational rearrangement of RNA molecules [32]. In this study, for in vitro use in a reporter refolding assay with EGFP, the fusion protein was first denatured with a chemical denaturant, and the fluorescence of EGFP was then measured in the absence and presence of TAR RNA. Furthermore, we found that label-free techniques, such as the His-tagging of R52 and R53Tat mutants selected as chaperna models using EGFP as a folding reporter, increased the folding ability of Tat. These results are interpreted as meaning that arginine is fairly resistant to mutations in the TAR RNA binding Tat protein in some studies, despite its wide application in protein folding, purification, and storage. The Tat–TAR interaction suggests that Tat is a molecular chaperone that prevents the aggregation of unfolded proteins. The proposed role of TAR involves ‘entropy transfer’ between Tat’s IDR and TAR 28, preventing R52Tat from misfolding and making it ‘folding competent’ (Figure 1) [38,76].

In conclusion, our study demonstrated that the net charge of the single-arginine mutant TatR52 in a cell-dense environment is associated with aggregation, as well as that the intermolecular electrostatic repulsion between charged residues in proteins affects protein refolding. These properties of RNA can stabilize intermediate folding and interfere with protein aggregation. This suggests that, although our understanding of the roles of various molecular chaperones is still limited, as the important role of virally encoded RNA in establishing proteome linkages is considered, there may be a variety of viral-encoding chaperones that have not yet been characterized [77,78].

These aspects could be extended to the supramolecular aggregates of genetically mutable viral diseases that have caused pandemics such as COVID-19, indicating that they should be considered for enhancing immunogenicity [79,80]. Therefore, our findings further enhance our understanding of the as-yet-unexplored viral-encoding chaperna that interacts with intracellular factors during cellular crowding [77]. The relative stability of the IDPs interacting with RNA-mediated folding and the therapeutic function of disease modulators linked to the development of drug-delivery applications need to be further investigated.

## 4. Materials and Methods

### 4.1. Cloning

The HIV Tat plasmid contained a 213-bp region. For PCR amplification, the primers used were Bam HI Tat forward 5′-GGATCC-ATGGAGCCAGTAGATCCTAGACTAGAG-3′ and EcoRI Tat reverse 5′-GAATTC-TTATTCGTGCCATTCGATTTTCTGAGCCTCGAAGATGTCGTTCAGACCTTCCTTCGGGCCTGTCGG-3′. BamH1 and EcoRI restriction sites were generated by inserting them into pcDNA3 using standard restriction cloning methods. The oligonucleotide forward primer was 5′-GGCACAAGCTGGAGTACAAC-3 and the reverse primer was 5′-ATGCCGTTCTTCTGCTTGTC-3′ for EGFP. A vector modified from pcDNA3 (Invitrogen, Carlsbad, CA, USA) was used for the construction of the expression vectors for the human wild-type Tat protein using standard restriction cloning methods. 

The Tat mutants used for replacing the single arginine (R) residues R52 and R53 with alanine (A) were produced by site-directed mutagenesis. The mutants and reporter-fused forms included Tat (R52A), Tat (R53A), Tat (R52A)-EGFP, and Tat (R53A)-EGFP. Tat (R52A) and Tat (R53A) were constructed by the PCR overlapping mutagenesis of the Tat gene. A hexa-histidine tag was added to the C-terminus of HIV Tat.

### 4.2. RNA Sequences

We used the following TAR RNA sequence for in vitro refolding: 5’- GGUCUC UCU GGU UAG ACC AGA UCU GAG CCU GGG AGC UCU CUG GCU AAC UAG GGA ACC -3′. Next, we performed RNase-free HPLC purification. TAR RNA was synthesized at a concentration of 100 nmol and with an RNA oligo length of 57 bp (M. BIOTECH Ltd., Egham, UK). 

### 4.3. Cell Culture

HeLa cells were grown in MEM (Modified Eagles Medium; WelGENE Inc., Daegu, Rep of Korea) supplemented with 10% FBS (Fetal bovine serum) and 1% penicillin/streptomycin in a 5% CO_2_ atmosphere at 37 °C. Cells were transfected when at 60–90% confluence using 2 µL of Lipofectamine 2000 (Invitrogen, Carlsbad, CA, USA) and 500 ng of plasmid per 2 × 10^6^ cells. The cells were washed once with growth medium, twice with 10× PBS at pH 7.4, and then once more with PBS alone. Transfections with the HIV Tat plasmid and the HIV Tat–GFP fusion plasmid were performed using Lipofectamine 2000 at 37 °C for 24 h. For the HIV Tat-GFP fusion plasmid and His-tagged HIV Tat plasmid protein expression, HeLa cells were lysed with FT Lysis Buffer containing 600 mM KCl, 20 mM Tris-Cl (pH 7.8), and 20% glycerol, as previously described [81]. The lysate was centrifuged at 15,000 RPM at 4 °C for 15 min; then, the obtained supernatant or 0.5–1 µg of lysate was used for the refolding assay or the biolayer light interferometry (BLI) assay, respectively.

### 4.4. Isolation of HIV Tat Protein from HeLa Cells

HeLa cells were lysed with FT Lysis Buffer containing 600 mM KCl, 20 mM Tris-Cl (pH 7.8), and 20% glycerol, as previously described [81]. The lysate was centrifuged at 15,000 rpm and 4 °C for 15 min; then, the obtained supernatant or 1 µg of lysate was used for the refolding assay.

### 4.5. Refolding of Fluorescence EGFP Reporter In Vitro

We expressed Tat fused with EGFP in mammalian cells. Tat-EGFP expression in cells was confirmed by laser-induced fluorescence using an inverted fluorescence microscope (Olympus IX71, Olympus, Tokyo, Japan). A 488 nm laser line was used for green fluorescence. The laser was focused into the channel using a 20× objective. The fluorescence signal was collected by the same lens and optically filtered. We detected the excitation/emission wavelengths of the green filter set U-MWIBA3 EGFP shifts (BP460-495; BA510-550). For the refolding EGFP reporter assay, the EGFP fluorescence emission spectra of the HIV Tat protein fused with EGFP in lysates from HeLa cells were measured in PBS buffer using the Chromo4™ System (Bio-Rad, Hercules, CA, USA). The EGFP reporter refolding assay measurements were performed at 30 °C. EGFP’s fluorescence kinetics were analyzed using the Opticon Monitor 3 (Bio-Rad). The multi-mode microplate reader FlexStation 3 system (Molecular Devices, LLC., San Jose, CA, USA) was also used for the detection of the EGFP fluorescence kinetics. The HIV Tat-EGFP fusion proteins harvested or isolated from HeLa cell lysates were denatured in 6 M guanidine-HCl and 1 mM DTT for 20 min at 40 °C. Next, the denatured proteins were diluted 25-fold in refolding buffer containing 50 mM Tris-HCl (pH 7.5), 50 mM NaCl, and 5 mM MgCl_2_.

### 4.6. Tat–BLI Assay In Vitro

BLI was performed using an Octet Red instrument (ForteBio, Pall Life Sciences, Fremont, CA, USA). The His Abs were immobilized on penta-His biosensors with varying concentrations of monomeric His-tagged Tat in HeLa lysates as the analyte in solution. The biosensors were hydrated for 10 min in PBS (pH 7.4), which was also used as a buffer in all the steps of the assay. Subsequently, the biosensors were first immersed in buffer for 120 s to establish a baseline and then in a solution containing 5 μg/mL of His Ab for 180 s to capture the Ab. After this, immersion in buffer was performed for a 60 s wash.

Next, the His Abs biosensors were immersed in a solution containing various concentrations of His-tagged Tat for 120 s to allow analyte/ligand association, followed by immersion in refolding buffer every 30 s for 8000 s. The analyte was serially diluted 2-fold from 500 to 50 nM, with each step being performed with 1000 rpm shaking in the Octet Red instrument (Sartorius, Göttingen, Germany). The binding affinity constants (K_D_; on-rate, kon; off-rate, koff) were determined using the Octet Analysis version 7 software (ForteBio, Pall Life Sciences). The fold changes for each mutant compared with the WT values were calculated. The BLI experiments were performed using the general BLI analysis protocol, as described previously [56]. Data analysis, including double reference subtraction, was performed with the ForteBio data analysis software.

## 5. Conclusions

In the intracellular environment, HIV Tat, an intrinsically disordered protein, is able to interact with multiple cellular proteins. However, studies on protein folding in crowded intracellular environments are scarce. Here, we explored the effects of intracellular molecular crowding on protein folding based on intracellular interactions, including TAT RNA as a chaperna-RNA that facilitates chaperone-function-mediated protein folding. 

In terms of HeLa cell crowding, we have demonstrated that TAR RNA allows chaperna function in a single-arginine mutant R52Tat using fused green fluorescence protein folding. The highly dynamic nature of the folding intermediates or intrinsically disordered regions (IDRs) of IDPs enables the detailed characterization of their structural rearrangement of the virally encoded TAR RNA, which keeps the mutant Tat protein ‘folding competent’, enabling its interaction with multiple cellular factors for transactivation. Our findings provide novel insight into the therapeutic potential of viral mutant gene encodings that modulate viral evolution-based chaperna linkages for RNA virus diseases.

## Data Availability

All the data in this paper are expressed as means, and the error bars indicate the standard deviations. For the visualization of the results and statistical analysis, we used the Graph Adobe CS7 software. UniProtKB—A6MI22 (A6MI22_9HIV1_*Human immunodeficiency virus 1*).

## Supplementary Materials

The following are available online at https://www.mdpi.com/article/10.3390/ijms22189998/s1.
