# Supplementary material for: TAR RNA Mediated Folding of a Single-Arginine-Mutant HIV-1 Tat Protein within HeLa Cells Experiencing Intracellular Crowding
Source: Int J Mol Sci. 2021 Sep 16;22(18):9998. doi: 10.3390/ijms22189998 (PMC8468913; doi:10.3390/ijms22189998)
Supplement: Supplementary file 1 [file ijms-22-09998-s001.zip › ijms-1315404-SI_0915.pdf]

Table S1. Enhancement factors for the refolding of the single-arginine-mutant Tat fusion EGFP.

DPI [1]	Refolding enhancement factor (%)	Relative of Refolded GFP intensity (L.U.)
wtTat-EGFP** / T[2]	I [3](L.U.)+TAR RNA/T (min)	I (0.3 μM TAR RNA) -I (0μM TAR RNA)
T 1 (20min)	0.013 *100% =1.3%	0.013 - 0.028 = - 0.067 (L.U.)
T 2 (30min)	0.017*100% =1.7%	0.017 - 0.042 = - 0.025 (L.U.)
T 3 (40min)	0.022*100% = 2.22%	0.022 - 0.049 = - 0.027 (L.U.)
R52Tat-EGFP / T	I (L.U.)+TAR RNA/T (min)	I (0.3 μM TAR RNA) -I (0μM TAR RNA)
T 1 (20min)	0.033*100% = 3.3%	0.033 - 0.031 = 0.002 (L.U.)
T 2 (30min)	0.042*100% = 4.2%	0.057 - 0.037 = 0.02 (L.U.)
T 3 (40min)	0.06*100% = 6%	0.06 - 0.04 = 0.02 (L.U.)
R53Tat-EGFP / T	I (L.U.)+TAR RNA/T (min)	I (0.3 μM TAR RNA) -I (0μM TAR RNA)
T 1 (20min)	0.0*100% = 0%	0.0 - 0.0 = 0 (L.U.)
T 2 (30min)	0.0*100% = 0%	0.0 - 0.0 = 0 (L.U.)
T 3 (40min)	0.0*100% = 0%	0.0 - 0.0 = 0(L.U.)
R52R53Tat-EGFP / T	I (L.U.)+TAR RNA/T (min)	I (0.3 μM TAR RNA) -I (0μM TAR RNA)
T 1 (20min)	0.0*100% = 0%	0.0 - 0.0 = 0 (L.U.)
T 2 (30min)	0.0*100% = 0%	0.0 - 0.0 = 0 (L.U.)
T 3 (40min)	0.01*100% = 1%	0.07 - 0.045 = 0.027 (L.U.)

[1] DPI ; Denatured Protein of Interest
[2] T ; Amount of Time
[3] I ; Saturated GFP intensity
** ; Protein of Interest (eg; wtTat-EGFP,R52Tat-EGFP … etc) was denatured at the same concentration from 0.05~0.1μg of native protein of interest.

Videos S1 and S2: This movie shows transmitted light time-lapse images of R52Tat–EGFP with TAR RNA in HeLa cells, some of which show the increase in green fluorescence owing to R52Tat–EGFP expression within and between the majority of cells. After the cotransfection of R52Tat–EGFP and TAR-RNA plasmids and 3 h of incubation, the regions of interest (ROIs) were traced for 2 h using differential interference contrast (DIC) microscopy with approximately 10 min of elapsed time per minute of movie (Audio Video Interleave; 10.06 MB and 10.06 MB for Videos S1 and S2).

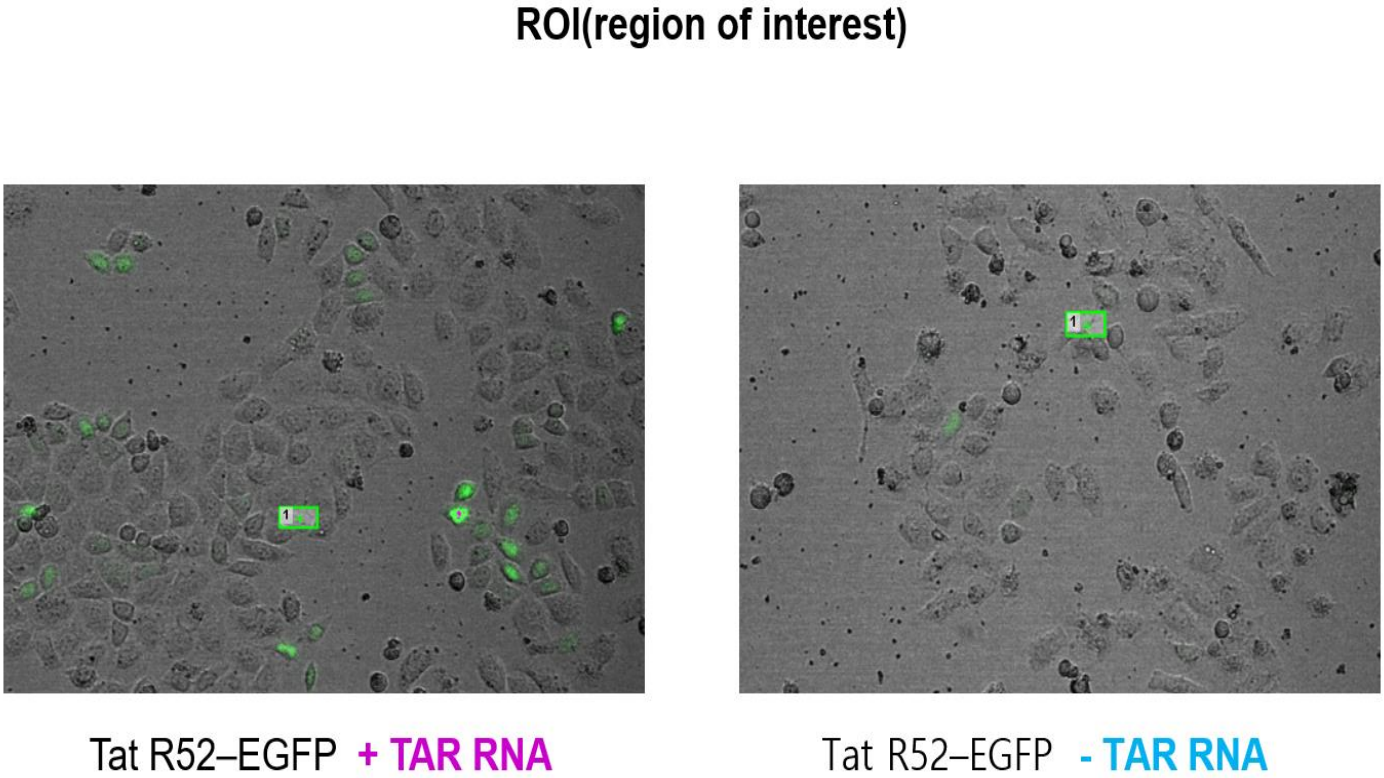

Figure S1. Time-lapse images of regions of interest (ROIs) for Tat–EGFP-expressing live HeLa cells. Representative merged image of green fluorescence and optical DIC (20×) for the comparison of the speed of folding of R52Tat–EGFP based on green fluorescence captured using a Nikon microscope (indicated by arrows) at 24 h.

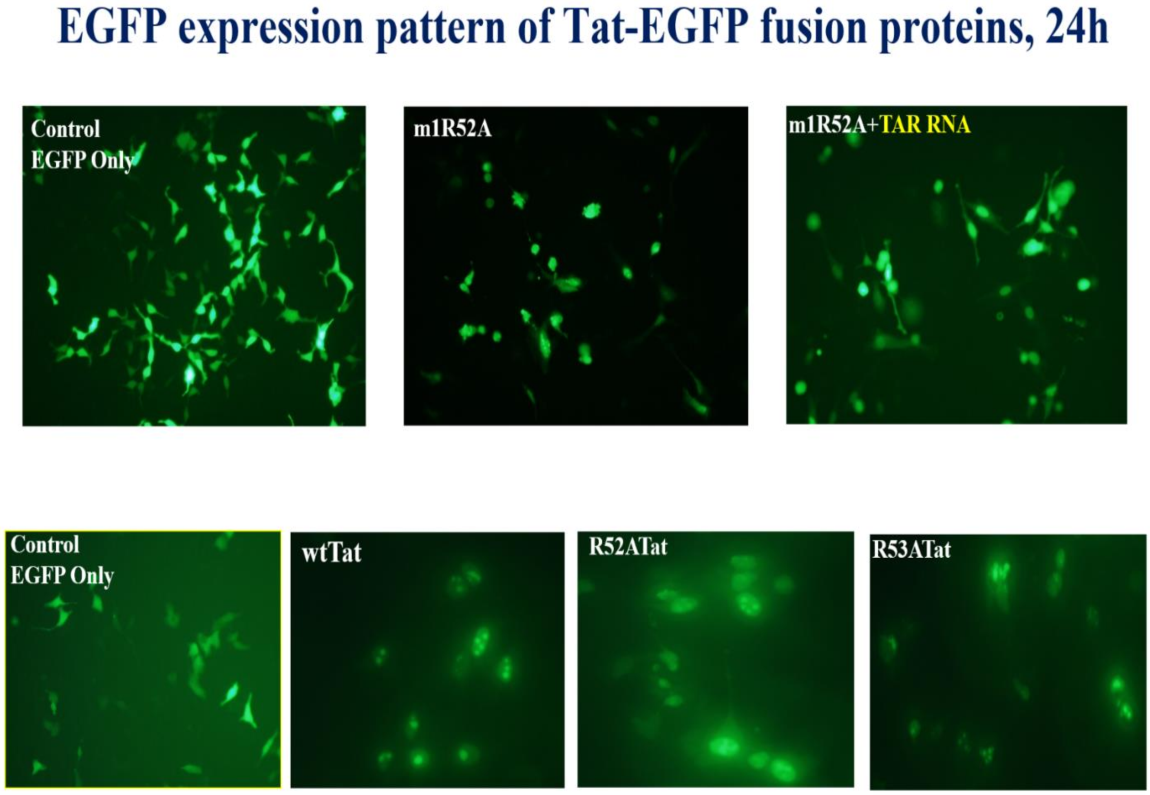

Figure S2. Treatment of Tat–EGFP-expressing HeLa cells with TAR RNA reduces the punctate localization in fluorescence images. The localization of EGFP folding reporter expression as a control and images of nuclear spots or nuclear accumulation were captured using a fluorescence microscope following treatment for 24 h. EGFP expression was detected by the fluorescence microscopy of the mutant Tat–EGFP fusion protein in HeLa cells and corresponds to nuclear accumulation. Nuclear accumulation was clearly observed for wild-type Tat–EGFP, R52Tat–EGFP, and R53Tat–EGFP expression but not for EGFP-only expression (control). The R52Tat–EGFP expression pattern following TAR-RNA treatment was similar to the EGFP-only expression pattern due to the reduced nuclear accumulation compared with samples that underwent no treatment. All the scale bars are 100 μm.

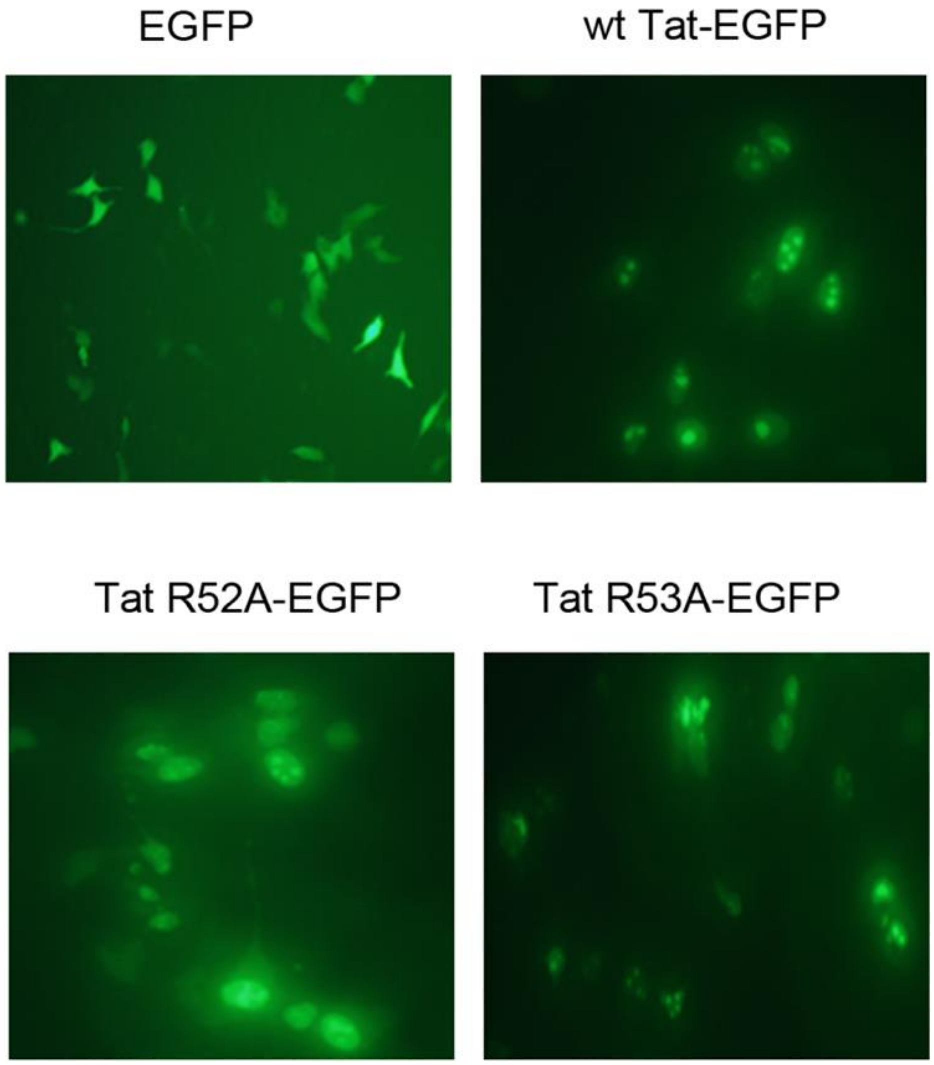

Figure S3. Subcellular localization of single-arginine-mutant Tat–EGFP in HeLa cells. EGFP expression in HeLa cells was detected using fluorescence microscopy. Expression corresponds to the expressed characteristics: EGFP (control), wild-type Tat–EGFP, R52Tat–EGFP, and R53Tat–EGFP. Images of EGFP localization and nuclear speckles or nuclear accumulation were captured using a fluorescence microscope. Fluorescent cells were transfected, and subcellular localization was shown using a GFP reporter. All the scale bars are 200 μm.

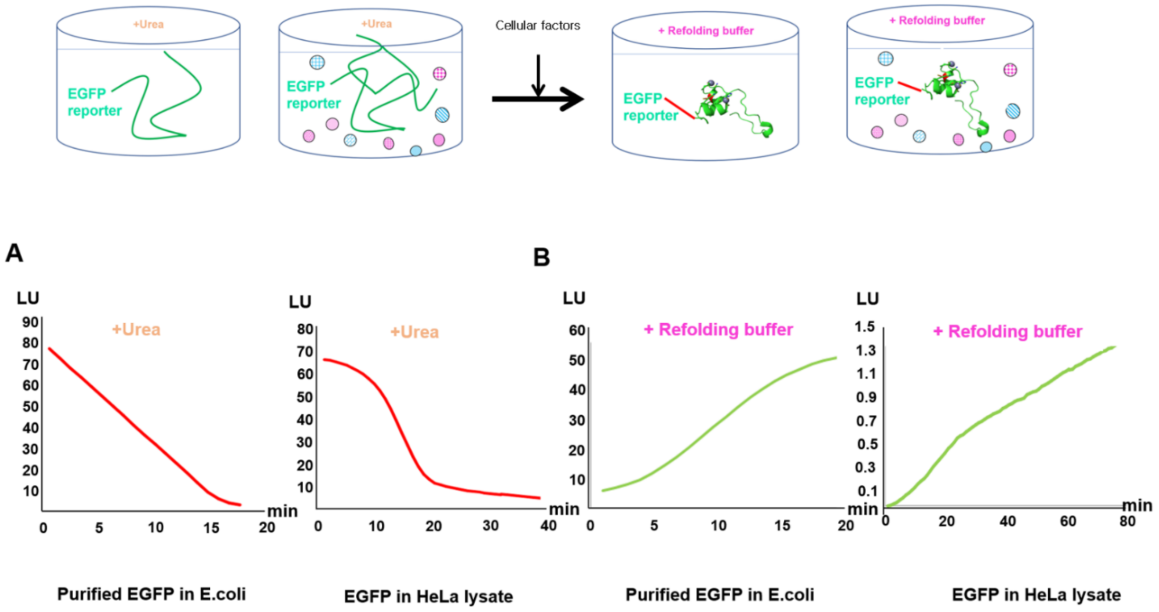

Figure S4. The refolding kinetic model showing the contribution of TAR RNA to EGFP folding in intracellularly crowded HeLa cells. The measurement of the fluorescence intensity for EGFP protein alone under denaturing or refolding buffer conditions. The refolding of EGFP only (green line) in HeLa lysate and EGFP only (green line) in *E. coli.* (**A**) Comparison of the effects of crowding or no crowding on denatured folding, which is reflected by the fluorescence of native EGFP, as measured under denaturing conditions at 1 min intervals for 20 min (*E. coli*) or 40 min (HeLa lysate). (**B**) Comparison of the effects of crowding or no crowding on refolding, which is reflected by the fluorescence of native EGFP, as measured under denaturing conditions at 1 min intervals for 20 min (*E. coli*) or 80 min (HeLa lysate).

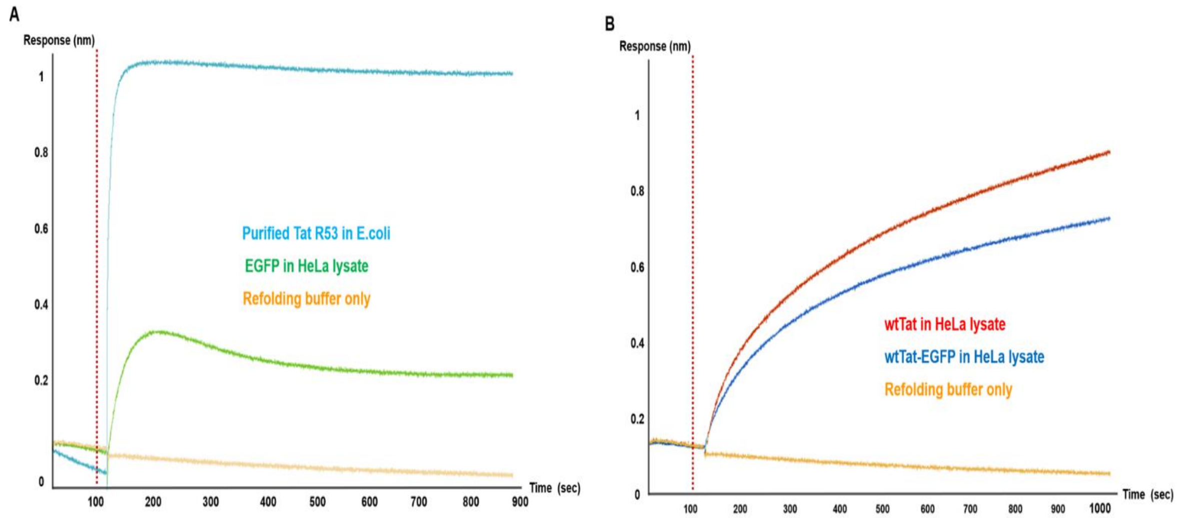

Figure S5. The kinetic refolding of the immobilized His-tagged Tat protein on an anti-penta-His biosensor tip without TAR RNA. Effects of the non-crowding (purified *E. coli*) of mutant R52Tat on the refolding yield in the absence of TAR RNA. Immobilized His-tagged wild-type (wt) Tat only and wtTat–EGFP refolding was assessed using an anti-penta-His biosensor in an Octect RED 96 system by monitoring the function of the chaperna without TAR RNA in HeLa lysates. (**A**) The refolding of EGFP in HeLa lysate (green), refolding buffer only (yellow), and non-crowded (purified *E. coli*) mutant R52Tat (blue). (**B**) Refolding of wtTat in HeLa lysate (red) and refolding buffer only (yellow) and crowding effect on wtTat–EGFP (blue) in HeLa lysates. Specific binding to anti-penta-H, left *Y*-axis. All the measurements are averages of duplicate experiments, and the error bars represent the data ranges. Buffer baselines were subtracted from all the measurements.
